# β-Glucan-Induced IL-10 Secretion by Monocytes Triggers Porcine NK Cell Cytotoxicity

**DOI:** 10.3389/fimmu.2021.634402

**Published:** 2021-02-19

**Authors:** Leen Hermans, Steffi De Pelsmaeker, Sofie Denaeghel, Eric Cox, Herman W. Favoreel, Bert Devriendt

**Affiliations:** Laboratory of Immunology, Department of Virology, Parasitology and Immunology, Faculty of Veterinary Medicine, Ghent University, Merelbeke, Belgium

**Keywords:** NK cells, IL-10, β-glucans, macrogard, curdlan, immune modulation, monocyte

## Abstract

Beta-glucans are naturally occurring polysaccharides present in cell walls of fungi, yeast, bacteria, cereals, seaweed, and algae. These microbe-associated molecular patterns (MAMPs) possess immunomodulatory properties. In human, it has been suggested that NK cells can be activated by β-glucans. Here, we aimed to elucidate whether β-glucans modulate porcine NK cell responses *in vitro* and if so, how these effects are mediated. We investigated the effect of two β-glucans, Macrogard and Curdlan, which differ in solubility and structure. Direct addition of β-glucans to purified porcine NK cells did not affect cytotoxicity of these cells against K562 target cells. However, when using PBMC instead of purified NK cells, β-glucan addition significantly increased NK cell-mediated cytotoxicity. This effect depended on factors secreted by CD14+ monocytes upon β-glucan priming. Further analysis showed that monocytes secrete TNF-α, IL-6, and IL-10 upon β-glucan addition. Of these, IL-10 turned out to play a critical role in β-glucan-triggered NK cell cytotoxicity, since depletion of IL-10 completely abrogated the β-glucan-induced increase in cytotoxicity. Furthermore, addition of recombinant IL-10 to purified NK cells was sufficient to enhance cytotoxicity. In conclusion, we show that β-glucans trigger IL-10 secretion by porcine monocytes, which in turn leads to increased NK cell cytotoxicity, and thereby identify IL-10 as a potent stimulus of porcine NK cell cytotoxicity.

**Graphical Abstract:**
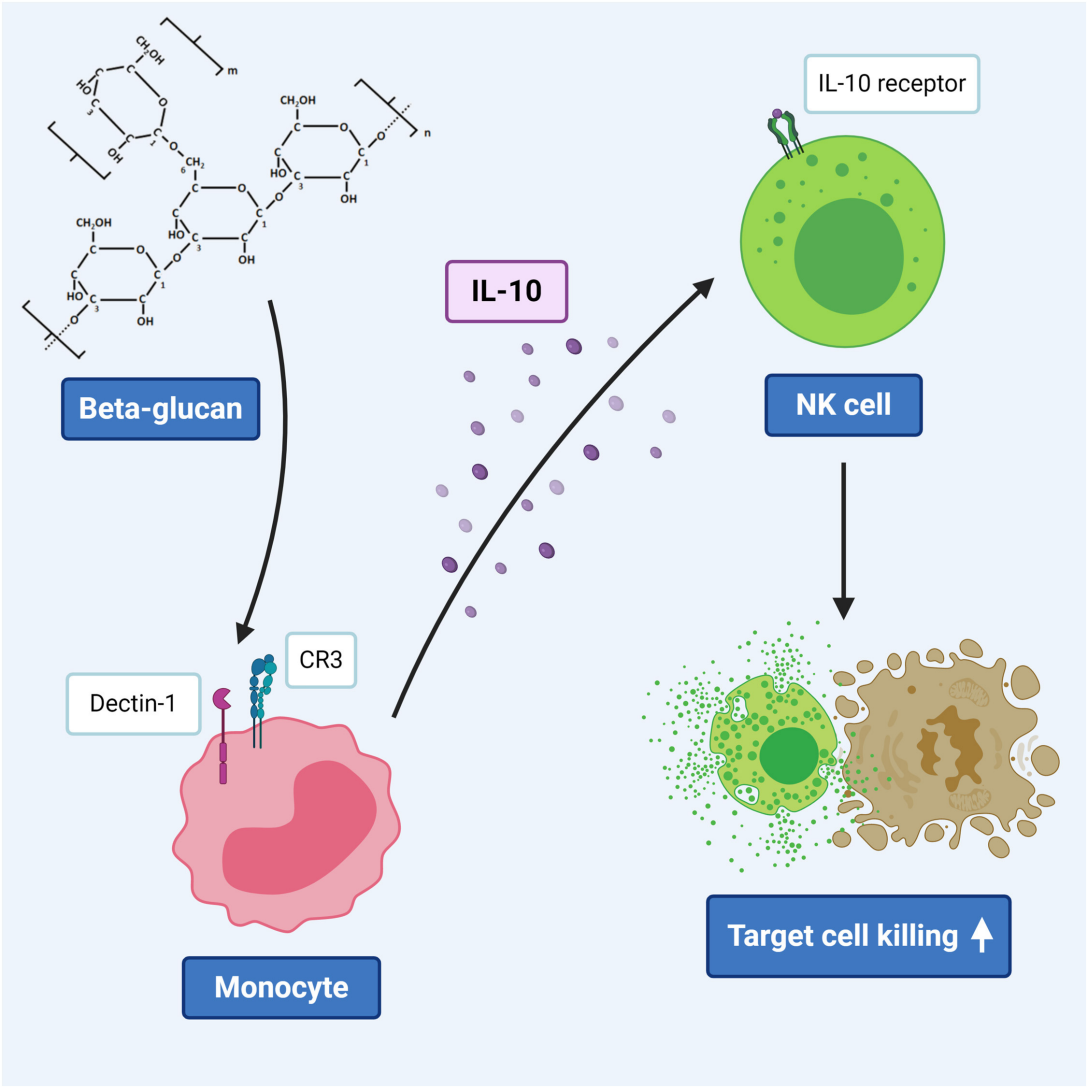
β-glucan induced IL-10 secretion by monocytes triggers porcine NK cell cytotoxicity.

## Introduction

Beta-glucans are naturally occurring glucose polymers that are important structural components of the cell wall of e.g., fungi, yeast, bacteria, cereals, seaweed, and algae (1). Thus, they originate from different sources and therefore are a heterogenous group, sharing a linear β-1,3-linked backbone structure, sometimes complemented with β-1,6-linked side chains. Other variable characteristics include branching frequency, length of the side branches, solubility, tertiary structure, and molecular weight. It is known that these variable features of β-glucans impact their immunomodulatory capacities (2). Recognition of β-glucans by the mammalian immune system occurs through pathogen-recognition receptors (PRR), such as dectin-1 and complement receptor 3 (CR3). Recognition of β-glucans and downstream signaling pathways were shown to be cell-type specific in humans (3) and mice (4), as well as in pigs (5). For instance, β-glucan-mediated effects in neutrophils occur mainly through recognition via the CR3 subunit CD11R3, whereas these effects in macrophages are mainly mediated by dectin-1 (3, 5, 6).

β-glucan-mediated effects have been studied in a wide variety of species (7). In mammals, they have been shown to possess important immunomodulatory capacities. For example, β-glucans enhance the phagocytotic capacity of macrophages (8) and oxidative burst of monocytes, macrophages and neutrophils (3, 9–11). They induce proliferation of peripheral blood mononuclear cells (PBMC) *in vitro* (11) and stimulate macrophages and dendritic cells to produce cytokines and chemokines, including TNF-α (11, 12), IL-12 (12), CXCL2, IL-6 (13), IL-1β, and IL-10 (11). β-glucans thus have the ability to trigger innate immune responses and shape innate and potentially also adaptive immunity (14). Therefore, β-glucans are also considered as interesting alternatives to prophylactic in-feed antibiotics in animal husbandry. Indeed, growth and immunity promoting antibiotics are banned by an increasing number of countries, including all European Union member states, and β-glucan-supplemented feed has been suggested to improve growth and overall health of commercially farmed pigs (15, 16).

Natural Killer (NK) cells are innate immune cells particularly known for their ability to kill virus-infected and malignant cells as well as to produce large amounts of IFN-γ upon stimulation (17). Activation of NK cells depends on the critical balance between inhibitory and activating signals transmitted by ligands on potential target cells (18). For example, MHC class I on the cell surface delivers a potent inhibitory signal to NK cells, and MHC class I downregulation on malignant cells or virus-infected cells therefore contributes to NK cell activation and killing of these target cells (19). This mechanism is also exploited to investigate NK cell-mediated cytotoxicity in *in vitro* assays, in which cell lines expressing limited amounts of MHC class I, such as K562, are co-cultured with effector cells.

It has been claimed that β-glucans can activate human NK cells, but conflicting results have been described in literature. One report described a directly enhanced NK cell-mediated cytotoxicity toward different target cells after preincubation of human NK cells with laminarin (20). Another study showed only an indirect effect on human NK cell cytotoxicity toward K562 cells, an NK cell-susceptible cancer cell line, after stimulation of PBMC with lentinan (21). Furthermore, inhibitory effects of β-glucans on NK cell activity have also been reported. For example, a dose-dependent inhibition of human NK cell killing of different target cells by zymosan and a particulate yeast glucan was described (22). In pigs, an *in vivo* study suggested that oral delivery of yeast-derived β-glucans impacted on the number of proliferating NK cells (23). This might indicate that β-glucans also influence porcine NK cells. Therefore, we set out to investigate whether two β-1,3-glucan preparations, differing in origin and structure, Macrogard (yeast-derived, β-1,6-branched glucan) and Curdlan (bacterial-derived, unbranched glucan), modulate porcine NK cell responses *in vitro*. Since administration of β-glucans *in vivo* is generally not accompanied by side effects (14), further insights into NK cell modulation by β-glucans may not only yield important information with regard to the use of β-glucans in animal husbandry, but also pave the way toward new strategies in vaccine development, anti-viral therapies and possibly cancer therapies.

## Materials and Methods

### β-Glucans

Macrogard was kindly provided by Biotec Pharmacon ASA (Tromsø, Norway) and Curdlan was purchased from Sigma (Bornem, Belgium). All material coming into contact with the β-glucans was made pyrogen-free. To this end, clean glassware and material was heated at 250°C for 4 h. β-glucan solutions were made in LAL reagent water (Catalog number W50-100; Lonza, Basel, Switzerland). Both types of β-glucan were solubilized via sonication. Quantification of endotoxin concentrations present in the β glucan preparations, was done by the Chromogenic Limulus Amebocyte Lysate (LAL) Test (Catalog number 50-647U, Lonza) and were 0.0362 EU/μg MG and 4.22 EU/μg CL.

### Isolation of PBMC

Heparinized blood samples (50 units/ml blood, LEO Pharma, Lier, Belgium) were obtained from the external jugular vein of pigs (8–29 weeks old) that were kept as blood donors at the Faculty of Veterinary Medicine, Merelbeke, Belgium. The blood sampling procedure was approved by the Ethical Committee of the Faculty of Veterinary Medicine (EC2017/121). PBMCs were isolated from whole blood by lymphoprep (Axis-Shield, Dundee, UK) density centrifugation. Red blood cells were lysed by a 10 min osmotic shock at room temperature by incubating the collected buffy coat in lysis buffer [composed of 90% NH4Cl 0.83% (w/v) and 10% TRIS 2.06% (w/v) in distilled water at a final pH of 7.2]. After a final washing step, PBMC were resuspended in 1 ml PBS-EDTA buffer and counted.

### Isolation of Porcine NK Cells

Primary porcine NK cells (CD3^−^CD172a^−^CD8α^+^) were isolated from porcine PBMC as described before (24) by negative MACS depletion of CD3^+^ and CD172a^+^ cells followed by FACS purification using monoclonal antibodies against porcine CD172a (IgG1, clone 74-22-15a), CD3 (IgG1, clone PPT3), and CD8α (IgG2a, clone 11/295/33) (25, 26). Secondary antibodies used were goat α-mouse IgG2a-AF647 (Catalog number A21241, Invitrogen, Carlsbad, CA, USA) and goat α-mouse IgG1-PE (Catalog number P21129, Invitrogen). Afterwards, porcine NK cells were purity sorted based on CD3^−^CD172a^−^CD8α^+^ expression using a BD FACS Aria III Cell Sorter (Beckman Coulter Biosciences, Franklin Lakes, NJ, USA), resulting in a ≥98% pure porcine NK population.

### Isolation and Cultivation of Porcine Monocytes

Freshly isolated PBMC were incubated with mouse anti-porcine CD14 antibodies (clone MIL-2, in-house production) or with mouse anti-porcine CD172a (IgG1, clone 74-22-15a) for 40 min. Cells were washed and secondary goat-anti-mouse IgG Microbeads (Catalog number 130-048-401, Miltenyi, Bergisch Gladbach, Germany) were added. After 20 min, cells were diluted in PBS-EDTA + 1% FCS and brought onto a LS column (Catalog number 130-042-401, Miltenyi) to generate CD14^+^/CD14^−^ and CD172a^+^/CD172a^−^ cell populations. Purity of all populations was checked by flow cytometry (Cytoflex; Beckman Coulter Biosciences): positive populations were >90% pure and neutrophil contamination was <5%, negative populations were >98% pure and neutrophil contamination was <1%. Afterwards, the obtained cell populations (PBMC, CD14^+^, CD14^−^, CD172a^+^, and CD172a^−^) were cultured in sterile 96-well-flat bottomed plates (Nunc, Thermo Fisher Scientific, Waltham, MA, USA) in Roswell Park Memorial Institute 1640 (RPMI) (Catalog number 52400-025, Thermo Fisher Scientific) supplemented with 10% (v/v) fetal calf serum (Thermo Fisher Scientific), 100 U/ml penicillin (Thermo Fisher Scientific), 100 μg/ml streptomycin (Thermo Fisher Scientific), and 2 mM L-glutamine [referred to as porcine NK medium (pNK medium)] with or without β-glucan supplementation (Macrogard or Curdlan at 10 μg/ml) for 16 h. PBMC, CD14^−^, and CD172a^−^ populations were cultured at 1 × 10^6^ cells per well (5 × 10^6^ cells/ml), whereas CD14^+^ and CD172a^+^ populations were cultured at 2 × 10^5^ cells per well (1 × 10^6^ cells/ml). After incubation, supernatant (SN) was collected and frozen at−80°C until later analysis (see section Indirect NK cell cytolytic assays and section ELISA).

### RT-PCR

Total RNA was isolated was isolated from freshly isolated PBMC and NK cells using the RNeasy mini kit (catalog number 74104; Qiagen, Hilden, Germany) according to the manufacturer's procedure. Concentration and purity of the RNA was measured by microvolume UV-Vis spectrophotometry (DeNovix, Wilmington, DE, USA). Afterwards, RNA was subjected to DNase treatment (RQ1 RNase-Free DNase; catalog number M6101; Promega, Madison, WI, USA) according to the manufacturer's protocol. Subsequently, RNA was reverse transcribed into cDNA using the SuperScript III Reverse Transcriptase kit (catalog number 18080093; Invitrogen) in the presence of a recombinant ribonuclease inhibitor (RNase OUT; catalog number 10777019; Invitrogen) according to the manufacturer's instructions to obtain cDNA in a single-step reaction consisting of 5 min at 25°C (priming), 50 min at 50°C (reverse transcription) and 15 min at 70°C (RT inhibition). The resulting cDNA served as a template for the polymerase chain reaction (PCR) assay. Primer oligonucleotides were synthesized by Integrated DNA Technologies (IDT, Coralville, IA). The sequences of the forward and reverse primers used for the amplification of the genes of interest and final primer concentrations can be found in Table 1. For non-preexisting primer oligonucleotide sequences, the primer design tool Primer-BLAST (NIH, USA) was used. The PCR reaction mix consisted of the Ready Mix (ReadyMix Taq PCR Reaction Mix with MgCl2, Catalog number P4600, Sigma-Aldrich, Saint Louis, MO, USA), sense and antisense primer and 100 ng of cDNA. The cycle conditions were 1 cycle of 10 min at 95°C followed by 40 cycles of 15 s at 95°C, 30 s at 60°C and 30 s at 70°C and finally 1 cycle of 10 min at 70°C. PCR products were visualized by GelRed (catalog number 41003, Biotium, Landing Parkway Fremont, CA, USA) agarose gel electrophoresis.

**Table 1:** Sequences and concentrations of primers used for target detection.

**Target**	**Accession no**.	**Primer sequence**	**Primer conc**.	**Ref**.
**β-actin**	AY550069	**Fw:** TCATCACCATCGGCAACG	250 nM	(27)
		**Rv:** TTCCTGATGTCCACGTCGC	250 nM	
**GAPDH**	AF017079	**Fw:** GGGCATGAACCATGAGAAGT	250 nM	(28)
		**Rv:** AAGCAGGGATGATGTTCTGG	250 nM	
**CYPA**	AY550069	**Fw:** CCCACCGTCTTCTTCGACAT	600 nM	(29)
		**Rv:** TCTGCTGTCTTTGGAACTTTGTCT	600 nM	
**Dectin-1**	XM_005655666.3	**Fw:** CAGCTCTCACAACCTCACCA	250 nM	–
		**Rv:** TGCCTTGATATAAATTCCAGCTCT	250 nM	
**TNF-α**	NM_214022	**Fw:** ACTGCACTTCGAGGTTATCGG	300 nM	(30)
		**Rv:** GGCGACGGGCTTATCTGA	300 nM	
**IL-6**	NM_214399	**Fw:** TTCACCTCTCCGGACAAAACTG	900 nM	(29)
		**Rv:** TCTGCCAGTACCTCCTTGCTGT	50 nM	
**IL-2**	NM_213861	**Fw:** AGCTCTGGAGGGAGTGCTAA	250 nM	–
		**Rv:** TCAAGTCAGTGTTGAGTAGATGCT	250 nM	
**IL-12 p40**	NM_213993	**Fw:** GGTTTCAGACCCGACGAACTCT	300 nM	(29)
		**Rv:** CATATGGCCACAATGGGAGATG	900 nM	
**IL-15**	NM_214390	**Fw:** TGGGCTGTATCAGTGCAGGT	250 nM	–
		**Rv:** TGGACTCTTGCAAAATGACGC	250 nM	
**IL-18**	NM_213997	**Fw:** TAGCTGAAAACGATGAAGACCTG	250 nM	–
		**Rv:** TGCCAGACCTCTAGTGAGGC	250 nM	
**IL-10**	NM_214041	**Fw:** AGGGTGTGCCCTATGGTGTTC	250 nM	–
		**Rv:** CGGGTGGGTAGGCTTGGAAT	250 nM	
**IL-10Rα**	XM_005656637.3	**Fw:** actgtgaccaacctcagcat	250 nM	–
		**Rv:** ggatcgttgaagaccaggacg	250 nM	
**IL-10Rβ**	NM_213771	**Fw:** GCAGTTACCGCCCAGTATGA	250 nM	–
		**Rv:** TGGGGGTGGTTTCGTCATTG	250 nM	

### Flow Cytometry

#### CR3 Cell Surface Expression

Freshly isolated porcine NK cells were washed with Dulbecco's Modified Eagle Medium (DMEM) (Thermo Fisher Scientific) + 1% fetal calf serum (FCS) (Thermo Fisher Scientific) and labeled with a primary mouse mAb for 20 minutes at 4°C. The following primary mAbs were used to identify the presence of CR3 on porcine NK cells: mouse anti-pig CD11R1 (clone MIL-4; Bio-Rad), mouse anti-pig CD11R3 (clone 2F4/11; Bio-Rad), and mouse anti-pig CD18a (clone PNK-I; Bio-Rad). Cells stained with isotype-matched irrelevant mAbs were used as a negative control. After incubation, cells were washed and stained with goat anti-mouse IgG1-PE. The cells were washed and Sytox blue (Catalog number S34857, Thermo Fisher Scientific) was added to discriminate live and dead cells. Doublets were excluded based on FSC-H/FSC-A and SSC-H/SSC-A and a minimal event count of 15,000 NK cells was used. Flow cytometry was performed using a Beckman Coulter Cytoflex and samples were analyzed using CytExpert software (Beckman Coulter).

#### Intracellular Cytokine Staining for IFN-γ

Freshly isolated PBMC (2.5 × 10^6^) were cultured in sterile 24-well-plates (Nunc, Thermo Fisher Scientific) in pNK medium with or without β-glucan supplementation (Macrogard or Curdlan at 10 μg/ml) or in the presence of a positive control cytokine-mix (IL-2: 20 ng/ml; IL-12: 25 ng/ml; IL-18: 100 ng/ml) for 16 h. Afterwards, Brefeldin A 1,000 × (BA) (Catalog number 00-4506-5, Invitrogen) was added to the cells for 4 h. Then, cells were collected and stained with Live/Dead Fixable violet Dead cell stain kit (Catalog number L34963, ThermoFisher Scientific) in the presence of BA for 30 min. Cells were washed and incubated with a mouse-anti-pig monoclonal anti-CD8α mAb (IgG2a, clone 11/295/33) in the presence of BA for 20 min. Cells were washed again and incubated with Dylight650 (Catalog number 84535, ThermoFisher Scientific) labeled purified mouse-anti-pig CD3 (IgG1, clone PPT3) and goat α-mouse IgG2a-AF488 (Catalog number A21131, Invitrogen) for 20 min in the presence of BA. Cells were washed and fixed and permeabilized using the Fixation/Permeabilization Solution Kit (Catalog number 554714, BD) according to the manufacturer's protocol. Finally, mouse-anti-human IFN-γ-PE Ab (IgG1) (Catalog number 502508, BioLegend, San Diego, CA, USA) or isotype control (Catalog number 400139, BioLegend) were added and incubated for 30 min. Cells were then washed and resuspended in PBS-EDTA and analyzed by FCM. A validated compensation matrix was applied. Doublets were excluded based on FSC-H/FSC-A and SSC-H/SSC-A, cells were selected based on their FSC-A/SSC-A properties and live/dead discrimination was performed (PB450-A/FSC-A). Within the PBMC population, NK cells were selected based on CD3-negative (APC-A/FSC-A) and CD8-positive (FITC-A/FSC-A) profile. Within the NK cell population, the percentage of IFN-γ producing cells (PE-A/FSC-A) was determined.

### Cytolytic Assays

#### Direct NK Cell Cytolytic Assays

Cytolytic assays were performed as described before (31). Briefly, freshly isolated porcine NK cells (1 × 10^6^) were cultured in sterile 96-well-flat bottomed plates (Nunc, Thermo Fisher Scientific) in porcine NK medium and primed with recombinant human (rh) IL-2 (20 ng/ml) (Catalog number PHC0026, Invitrogen) for 16 h. Afterwards, IL-2 primed NK cells were collected and transferred to a sterile conical bottomed 96-well-plate (Nunc, Thermo Fisher Scientific) at a density of 3 × 10^5^ cells per well and cultured for 2 h in porcine NK medium with or without β-glucan supplementation (Macrogard or Curdlan at 10 μg/ml). K562 cells, used as target cells, were cultured in IMDM (Catalog number 12440-053, Thermo Fisher Scientific) supplemented with 10% FCS, 100 U/ml penicillin, 100 μg/ml streptomycin and 0.05 mg/ml gentamycin. Target cells were labeled with carboxyfluorescein succinimidyl ester (CFSE) dye (Catalog number C34571, Invitrogen) according to the manufacturer's recommendations. After 2 h of β-glucan priming, target cells were co-incubated with the NK cells at an effector:target ratio of 25:1 for 4 h at 37°C. Thereafter, viability of target cells was assessed by propidium iodide (Catalog number BMS500PI, Invitrogen) staining and flow cytometric analysis.

#### Cytolytic Assay: Gating Strategy

Samples were analyzed using a Cytoflex (Beckman Coulter). Doublet discrimination was carried out based on FSC-H/FSC-A and SSC-H/SSC-A and cells were selected based on their FSC-A/SSC-A properties. CFSE-labeled target cells were selected on FSC-A/FITC-A, after which CFSE-positive cells were gated on cell viability on FSC-A/PC5.5-A. For all conditions 5,000 CFSE-labeled target cells were measured and the percentage of NK mediated lysis was calculated using the formula (% dead target_NK_ – % dead target_spont_)/(%dead target_max_ – % dead target_spont_).

#### PBMC Cytolytic Assays

Freshly isolated PBMC (1 × 10^6^) were cultured in sterile 96-well-flat bottomed plates (Nunc, Thermo Fisher Scientific) in porcine NK medium with or without β-glucan supplementation (Macrogard or Curdlan at 10 μg/ml) for 16 h. Afterwards, SN was collected and frozen at−80°C until later analysis (see section Indirect NK cell cytolytic assays and section Depletion of cytokines via immunoprecipitation). Next, PBMC were collected and transferred to a sterile conical bottomed 96-well-plate (Nunc, Thermo Fisher Scientific) at a density of 1.2 × 10^6^ cells per well and cultured in porcine NK medium with or without β-glucan supplementation (Macrogard or Curdlan at 10 μg/ml) for 2 h. Afterwards, CFSE-labeled K562 target cells were added at an effector:target ratio of 100:1 for 4 h at 37°C. Discrimination between live/dead cells and resulting analysis was performed as described in section Cytolytic assay: gating strategy.

#### Indirect NK Cell Cytolytic Assays

SN collected after 16 h culture of PBMC (see section PBMC cytolytic assays) or different subpopulations (see section isolation and cultivation of monocytes) with or without β-glucan supplementation or immunoprecipitated SN (see section depletion of cytokines via immunoprecipitation) was stored at −80°C until further analysis. At the moment of analysis, the SN was added to freshly isolated porcine NK cells for 16 h. Afterwards, NK cells were washed and resuspended in fresh porcine NK medium and CFSE-labeled target cells were added at an effector:target ratio of 25:1 for 4 h at 37°C. Discrimination between live/dead cells and resulting analysis was performed as described in section Cytolytic assay: gating strategy.

### Cytokine Production

#### qPCR

Freshly isolated PBMC (30 × 10^6^) were cultured in 6-well-plates (Nunc, Thermo Fisher Scientific) in porcine NK medium with or without β-glucan supplementation (Macrogard or Curdlan at 10 μg/ml) at 37°C for 4 h. After 4 h, cells were collected and total RNA was isolated using the RNeasy mini kit (catalog number 74104; Qiagen) according to the manufacturer's procedure. Quantification of RNA and reverse transcription was performed as described in section RT-PCR and the resulting cDNA served as a template for the quantitative polymerase chain reaction (qPCR) assay. Primer oligonucleotides were designed and synthesized as described in section RT-PCR. A SYBR green PCR master mix (catalog number 4309155; Applied Biosystems, Thermo Fisher Scientific) was used for quantitative PCRs (qPCRs) using 1 μl (50 ng) of the cDNA template resulting from RT and sense and antisense primer, following the protocol provided by the manufacturer. qPCRs were performed in a final volume of 20 μl on MicroAmp Fast optical 96-well-reaction plates (catalog number 4346906; Applied Biosystems, Thermo Fisher Scientific) using a StepOnePlus real-time PCR system (catalog number 4376600; Applied Biosystems, Thermo Fisher Scientific). The cycle conditions were 1 cycle of 10 min at 95°C and 40 cycles of 15 s at 95°C, 30 s at the annealing temperature (60°C), and 30 s at 72°C. The relative expression levels (RQ) of the target genes was analyzed by the double delta threshold cycle method and normalized to the level of expression of the reference genes [β-actin, GAPDH and Cyclophilin A (CYPA)] and to the control condition (Medium). Reference genes were selected based on geNorm analysis using qBase+ software.

#### ELISA

The concentrations of TNF-α, IL-6, IL-10, and IL-12/-23 were measured in culture SN using commercially available porcine-specific ELISA kits (TNF-α, IL-6, IL12-/IL-23 p40: R&D Systems Inc.; Minneapolis, MN, USA; IL-10: Invitrogen, Porcine IL-10 Antibody Pair Kit) according to the manufacturer's recommended protocols.

### Stimulation of NK Cells With Recombinant Proteins

Freshly isolated porcine NK cells (3 × 10^5^) were cultured in sterile 96-well V-bottomed plates (Nunc, Thermo Fisher Scientific) in porcine NK medium with or without supplementation of purified recombinant porcine TNF-α (R&D systems, #690-PT), IL-6 (R&D systems, #686-PI), or IL-10 (R&D systems, #693-PI) for 16 h. Afterwards, CFSE-labeled K562 target cells were added at an effector:target ratio of 25:1 for 4 h at 37°C. Discrimination between live/dead cells and resulting analysis was performed as described in section Cytolytic assay: gating strategy.

### Depletion of Cytokines via Immunoprecipitation

Freshly isolated PBMC (1 × 10^6^) were cultured in sterile 96-well-flat bottomed plates (Nunc, Thermo Fisher Scientific) in porcine NK medium with or without β-glucan supplementation (Macrogard or Curdlan at 10 μg/ml) for 16 h. Afterwards, SN was collected and frozen at−80°C and used for immunoprecipitation of TNF-α, IL-6, or IL-10 afterwards. Briefly, 400 μl of SN was incubated in the presence of an antibody specific for TNF-α, IL-10, or IL-6 (listed in Table 2) or without antibody as negative control for 4 h at 4°C on a rotating wheel. Meanwhile, protein A/G magnetic beads (Catalog number 88802, Thermo Fisher) were washed according to the manufacturer's recommended protocol. Thereafter, the incubated SN was added to a 1.5 mL microcentrifuge tube containing pre-washed magnetic beads and incubated for 4 h at 4°C on a rotating wheel. Finally, beads were removed with a magnetic stand and the SN was collected and stored at −80°C. SN was analyzed for immunoprecipitation efficiency with commercially available porcine-specific ELISA kit (see section ELISA) and used for CA (see section Indirect NK cell cytolytic assays).

**Table 2:** Antibodies used for immunoprecipitation.

**Target**	**Supplier**	**Catalog number**
TNF-α	R&D systems	MAB6903
IL-6	R&D systems	686-PI-025
IL-10	R&D systems	MAB6931

### Statistical Analysis

Statistical analysis was performed using Graphpad Prism 6. Data were analyzed for statistical differences with a Friedman test (in the case of one independent variable) and two-way ANOVA (in the case of two independent variables) at the 5% significance level. *Post hoc* analysis was performed using Dunn's test and Sidak's test, respectively. The synergistic effect of LPS on IL-10 secretion by monocytes upon Macrogard stimulation was tested for statistical significance using a one-tailed paired *t*-test.

## Results

### β-Glucans Are Unable to Directly Stimulate Porcine NK Cell Cytotoxicity

**Figure 1:**
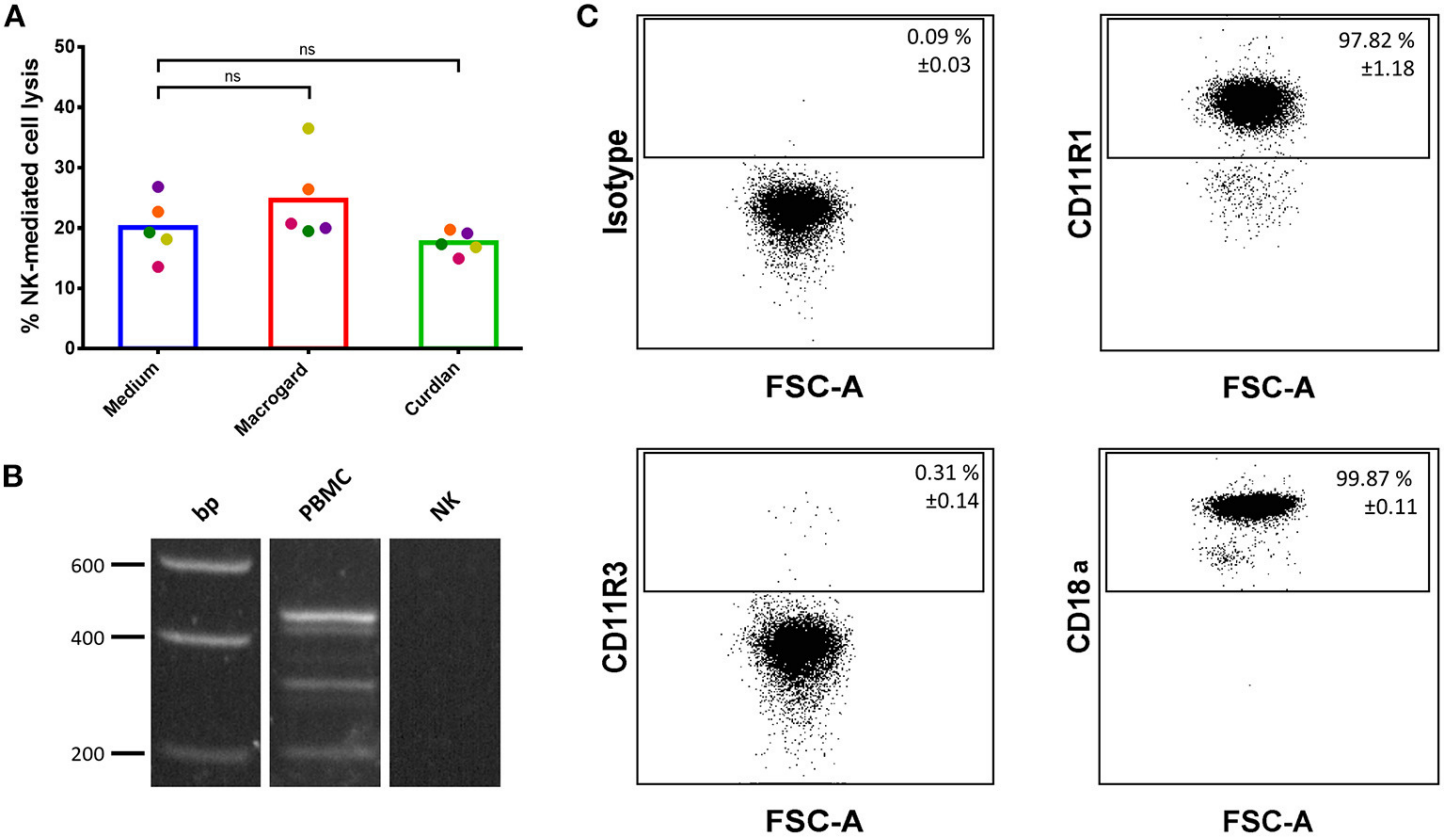
β-glucans are unable to directly activate porcine NK cells. **(A)** Purified IL-2-primed primary porcine NK cells were cultured for 2 h with or without β-glucan (Macrogard or Curdlan at 10 μg/ml). Afterwards, primed NK cells were co-cultured with K562 target cells for 4 h to assess cytotoxic activity. **(B)** RT-PCR analysis of dectin-1 isoform expression on PBMC and porcine NK cells. Isoform A was detected at 450 bp, isoform B at 312 bp and the intracellular isoform E at 213 bp as well as some other uncharacterized isoforms on PBMCs but not on NK cells. **(C)** Flow cytometric analysis of CR3 subunit expression (CD11R1, CD11R3, and CD18a) on porcine NK cells. Dot plots are representative for three animals. Data in the top right corner of each dot plot are mean ± SD values (*n* = 3). ns, not significant.

First, the ability of the β-glucans Macrogard and Curdlan to directly stimulate NK cell cytotoxic activity was investigated. Results showed that Macrogard nor Curdlan at 10 μg/ml were able to modulate the cytotoxic capacity of IL-2 primed NK cells against NK-susceptible K562 cells (Figure 1A). In addition, further increasing the concentration of these β-glucans (20, 50, or 200 μg/ml) also did not affect the cytolytic activity of IL-2 primed NK cells (Supplementary Figure 1).

To understand the reason for the lack of response of NK cells toward β-glucans, the expression of the two major β-glucan receptors, dectin-1, and Complement Receptor 3 (CR3), on porcine NK cells was investigated. Unfortunately, neither porcine-specific nor human cross-reactive anti-dectin-1 antibodies are available, hampering flow cytometric analysis of dectin-1 expression on porcine cells (32). In addition, different dectin-1 isoforms have been described in pigs (33), further complicating antibody-mediated detection of dectin-1, as such an approach may possibly miss certain isoforms. Therefore, we opted for mRNA detection via RT-PCR and primers that recognize these isoforms were designed. As shown in Figure 1B, dectin-1 mRNA was not detectable in freshly isolated porcine NK cells, while PBMCs express different isoforms of this β-glucan receptor. Expression of CR3 on porcine NK cells was investigated by flow cytometry. In pigs, this β_2_-integrin is composed of an alpha-subunit (CD11R1 or CD11R3) and a beta-subunit (CD18a). A functional study in pigs showed that recognition of β-glucans by CR3 solely depends on CD11R3 and is independent of CD11R1. As displayed in Figure 1C, this analysis revealed that most NK cells express CD11R1 (97.82% ± 1.18) in combination with the β-subunit CD18a (99.87% ± 0.11). The β-glucan recognizing subunit CD11R3 (0.31% ± 0.11), on the other hand, was only present on a very minor subset of NK cells. We therefore conclude that porcine NK cells do not or do only minimally express either of the two major β-glucan receptors, in line with the observed lack of a direct effect of β-glucan addition on NK cell cytotoxicity.

### β-Glucans Indirectly Stimulate NK Cell Cytotoxicity via Monocytes

**Figure 2:**
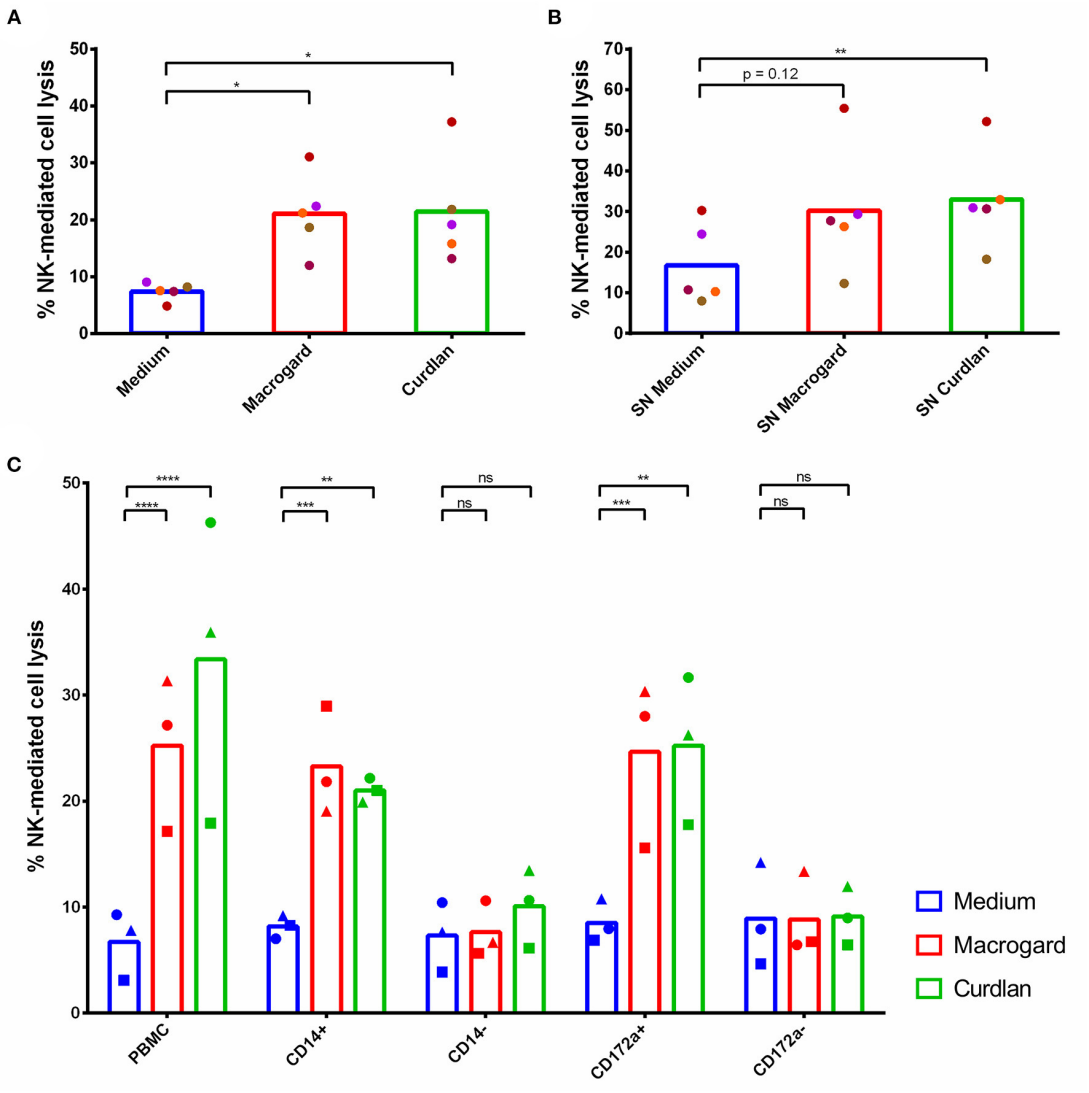
β-glucans indirectly stimulate NK cell cytotoxicity via monocytes. **(A)** PBMC were cultured for 16 h with or without β-glucans (Macrogard or Curdlan at 10 μg/ml). Afterwards, primed PBMC were collected and co-cultured with K562 target cells for 4 h to assess cytotoxic activity. **(B)** Supernatant of PBMC cultured for 16 h with or without β-glucans (Macrogard or Curdlan at 10 μg/ml), was collected and added to freshly isolated porcine NK cells for 16 h. Afterwards, primed NK cells were co-cultured with K562 target cells for 4 h to assess cytotoxic activity. **(C)** Supernatant of PBMC, CD14^+^, CD14^−^, CD172a^+^, and CD172a^−^ populations, cultured for 16 h with or without β-glucan (Macrogard or Curdlan at 10 μg/ml), was collected and added to freshly isolated porcine NK cells for 16 h. Afterwards, primed NK cells were co-cultured with K562 target cells for 4 h to assess cytotoxic activity. *p < 0.05; **p < 0.01; ***p < 0.001; ****p < 0.0001; ns, not significant.

It is well-established that addition of β-glucans to PBMC may result in a variety of responses, including secretion of cytokines, which in turn may lead to modulation of other immune cells. To investigate whether such downstream effects may affect NK cell cytotoxicity, PBMC were primed overnight with the β-glucans Macrogard and Curdlan and the cytotoxicity of the PBMC toward K562 cells was analyzed. Cytolytic assays showed a statistically significant increased NK-mediated lysis of target cells by PBMC primed with β-glucans (Figure 2A). This increase in cytotoxic activity against K562 cells was abrogated when NK cells were removed from the PBMC population via FACS (Supplementary Figure 2), confirming that cytotoxicity was indeed exerted by NK cells. Although NK cells are capable of producing large amounts of IFN-γ upon activation, intracellular cytokine staining analyzed by flowcytometry showed that no detectable levels of IFN-γ were produced by NK cells in β-glucan-treated PBMC (Supplementary Figure 3).

To further investigate whether the activating effect of β-glucan-treated PBMC on NK cell cytotoxicity was mediated by secreted molecules or depended on cell-cell contacts, the supernatant (SN) of β-glucan primed PBMC was collected and added to freshly isolated and purified NK cells. After overnight incubation with the SN, NK cells showed an enhanced cytolytic activity toward K562 cells (Figure 2B), indicating that the NK cell-activating factors present in β-glucan-primed PBMC consist of secreted molecules, likely cytokines.

To identify the cell type responsible for the production of these secreted molecules, PBMC were MACS-sorted into CD14^+^ and CD14^−^ cell fractions and stimulated overnight with either Macrogard or Curdlan. In porcine PBMC, CD14 is solely present on monocytes and a small subset of B cells, and absent from e.g., dendritic cells (34–36). After β-glucan priming of the different populations, SN was collected and incubated overnight with freshly isolated NK cells to analyze their potential to affect NK cell cytotoxic activity. The results showed that the β-glucan primed CD14^+^ population secreted factors that enhance the cytolytic activity of porcine NK cells (Figure 2C). Depletion of CD14^+^ cells from the PBMC fraction (=CD14^−^) completely abrogated the indirect stimulatory effect of β-glucans on porcine NK cell cytotoxicity. These results point to monocytes as being both essential and sufficient to trigger NK cell-activating factors upon β-glucan priming of PBMC. To assure that the observed effects using CD14^+^ populations were not in part mediated by the small population of CD14^+^ B cells, these assays were repeated with CD172a^+^ and CD172a^−^ cell populations. CD172a is expressed by monocytes and dendritic cells, but not by B cells (34, 37). Upon stimulation of NK cells with SN of β-glucan-primed CD172a^−^ cells, no stimulation of NK cell cytotoxicity was observed. In contrast, priming of NK cells with SN of β-glucan-primed CD172a^+^ cells increased the NK cell cytotoxicity to similar levels as seen with SN from the CD14^+^ population (Figure 2C). Therefore, we conclude that monocytes play a cardinal role in the production of secreted molecules that increase NK cell cytotoxicity upon β-glucan priming of PBMC.

### IL-10 Is Responsible for Stimulation of NK Cell Cytotoxicity Upon β-Glucan Priming of Monocytes

**Figure 3:**
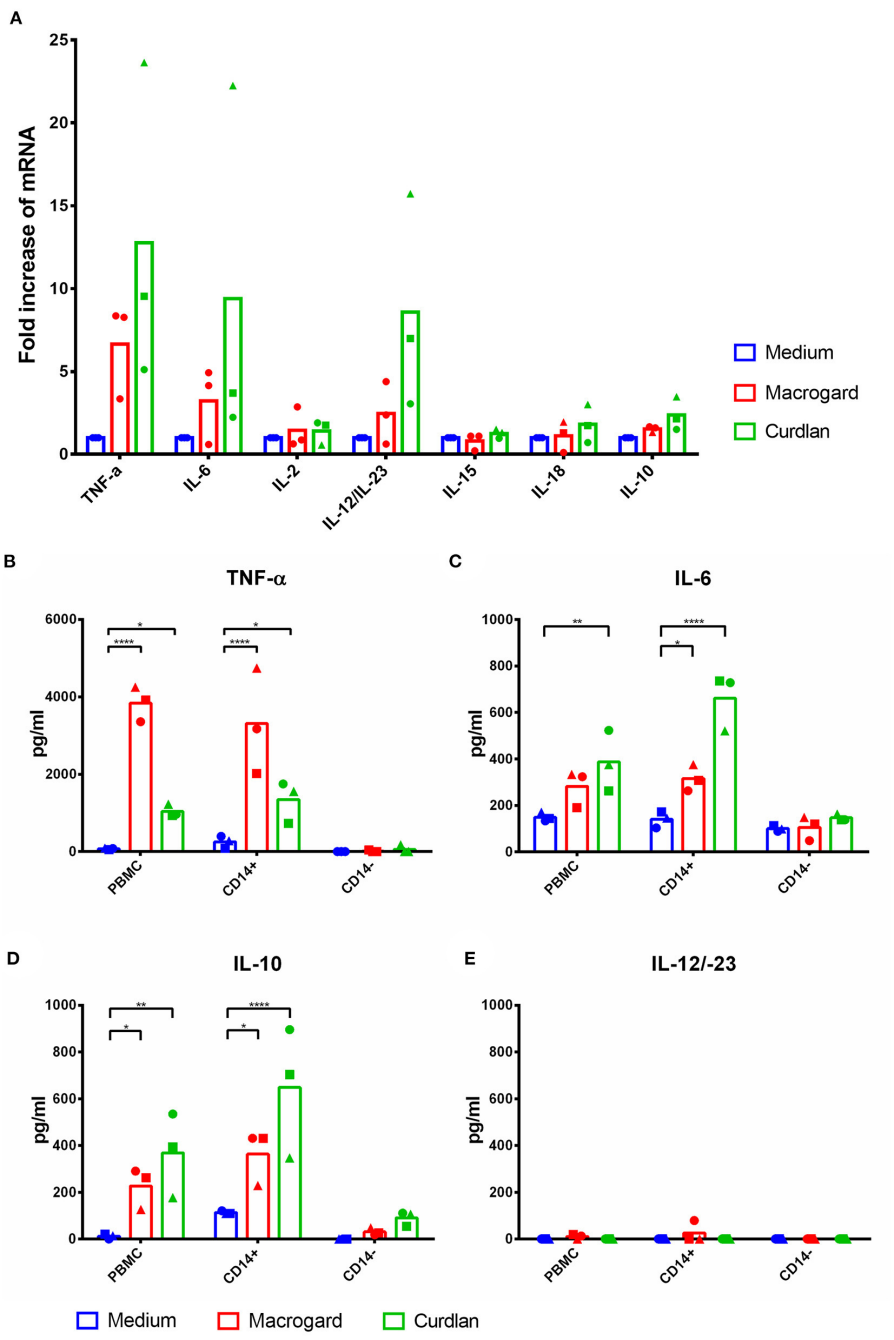
TNF-α, IL-6, and IL-10 are upregulated in PBMC and monocytes after β-glucan stimulation. **(A)** PBMC were cultured with or without β-glucan (Macrogard or Curdlan at 10 μg/ml). After 4 h, primed PBMC were collected and used for qPCR analysis. **(B–E)** PBMC, CD14^+^, and CD14^−^ populations were cultured with or without β-glucan (Macrogard or Curdlan at 10 μg/ml) for 16 h. Culture supernatant was collected and analyzed by ELISA for presence of cytokines. *p < 0.05; **p < 0.01; ****p < 0.0001.

To investigate which cytokines were possibly involved in the observed activation of NK cell cytotoxicity, mRNA expression levels of TNF-α, IL-6, IL-2, IL-12p40, IL-15, IL-18, and IL-10 were investigated in β-glucan-stimulated PBMC (4 h) by RT-qPCR. Results showed that TNF-α, IL-6, and IL-12p40 were upregulated upon β-glucan stimulation (Figure 3A). Although expression levels of IL-10 were not substantially upregulated, previous research has shown that IL-10 at the protein level is significantly upregulated upon β-glucan stimulation of PBMC (11). Therefore, we decided to include IL-10 in our further analysis at the protein level by ELISA. An upregulation at the protein level of TNF-α, IL-6, and IL-10 was confirmed, whereas no increase of IL-12p40 protein was detected upon β-glucan stimulation of PBMC or CD14+ cells (Figures 3B–E).

**Figure 4:**
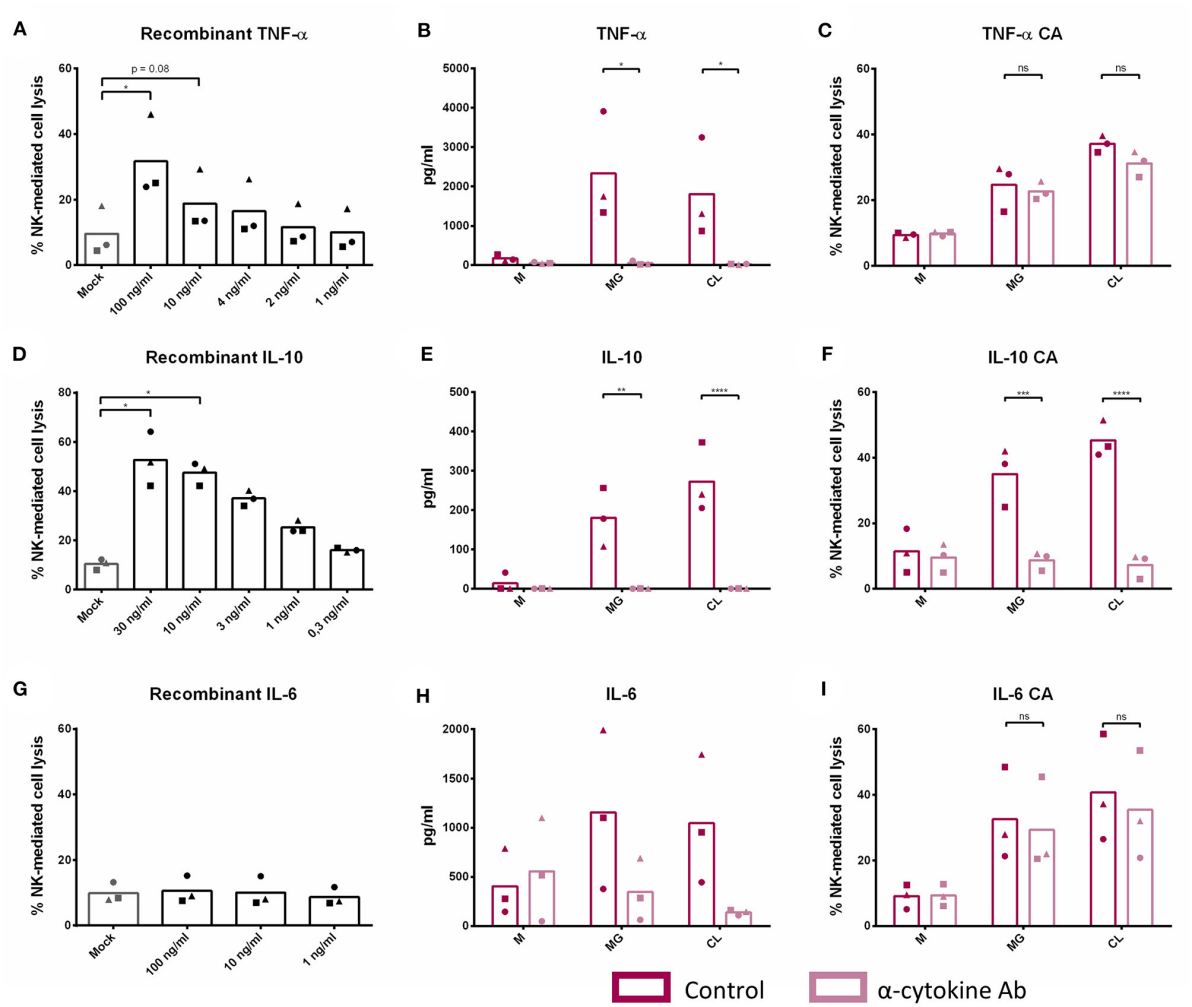
IL-10 plays a cardinal role in the indirect stimulation of NK cell cytotoxic capacity upon β-glucan priming of monocytes. Freshly isolated NK cells were cultured with recombinant porcine TNF-α **(A)**, IL-10 **(D)**, and IL-6 **(G)** at different concentrations for 16 h. Afterwards, primed NK cells were co-cultured with K562 target cells for 4 h to assess cytotoxic activity. PBMC were cultured with or without β-glucan (Macrogard or Curdlan at 10 μg/ml) for 16 h. Afterwards, supernatant was collected and used for immunoprecipitation (IP) with a specific anti-cytokine antibody for TNF-α, IL-10, or IL-6 or without antibody as negative control. Afterwards, efficiency of IP was controlled by ELISA **(B,E,H)**. Supernatant obtained after IP was added to freshly isolated NK cells to define the role of TNF-α **(C)**, IL-10 **(F)**, and IL-6 **(I)** in the indirect stimulation of NK cells upon β-glucan priming of monocytes. After 16 h of incubation, primed NK cells were co-cultured with K562 target cells for 4 h to assess cytotoxic activity. *p < 0.05; **p < 0.01; ***p < 0.001; ****p < 0.0001; ns, not significant.

**Figure 5:**
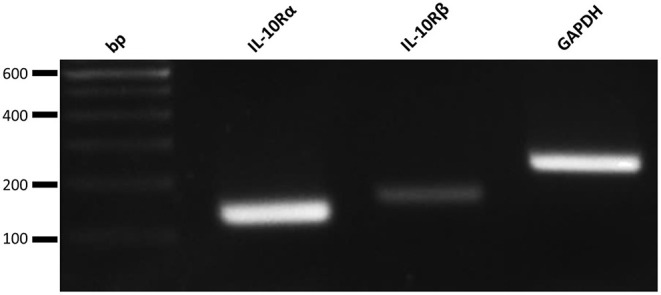
Freshly isolated porcine NK cells do express the IL-10 Receptor subunits alpha (IL-10Rα) and beta (IL-10Rβ). RT-PCR analysis of both subunits of the IL-10 receptor and a positive control (GAPDH).

To analyze the potential contribution of each of the upregulated cytokine proteins (TNF-α, IL-6, and IL-10) in the increased NK cell mediated cytotoxicity observed upon β-glucan stimulation of PBMC, NK cell cytotoxicity assays were performed. First, the effects of recombinant porcine TNF-α, IL-10, or IL-6 on cytotoxicity of freshly isolated pNK cells were investigated. Both recombinant TNF-α (Figure 4A) and IL-10 (Figure 4D) were able to directly stimulate NK cell cytotoxicity in a concentration-dependent manner, whereas recombinant IL-6 (Figure 4G) did not modulate NK cell cytotoxicity. To further assess the role of these cytokines in activating NK cell cytotoxicity, TNF-α, IL-10, and IL-6 were depleted by immunoprecipitation from the SN of β-glucan-primed PBMC (Figures 4B,E,H, respectively). Upon stimulation of NK cells with IL-10-depleted SN, we observed a complete abrogation of the β-glucan-triggered increase in NK cell cytolytic activity (Figure 4F), while depletion of TNF-α (Figure 4C) or IL-6 (Figure 4I) did not alter NK cell cytotoxicity. Since involvement of LPS, present in bacterial-derived CL, in IL-10 secretion by PBMC could not be completely excluded, we determined IL-10 levels in supernatant of PBMC stimulated with 10 ng/ml LPS, corresponding to the upper level of endotoxin concentration found in CL. The results (Supplementary Figure 4) show that LPS alone induces only minimal levels of IL-10 secretion by PBMC, whereas PBMC stimulated with MG do secrete substantial levels of IL-10. When MG is supplemented with LPS (10 ng/ml), a synergistic effect on IL-10 production by PBMC was observed. We therefore conclude that the β-glucan structure of MG alone is able to induce IL-10 production by PBMC, but that LPS is able to synergistically enhance this response. This might explain the slightly larger cytotoxic response of NK cells that is generally seen after stimulation with bacterial-derived CL compared to yeast-derived MG. To further confirm the role of IL-10 in the β-glucan mediated NK cell activation, expression of both the alfa- and beta-subunit of the IL-10 receptor by porcine NK cells was confirmed by RT-PCR (Figure 5). These results not only highlight a pivotal role for IL-10 in the stimulation of NK cell cytotoxic capacity upon β-glucan priming of monocytes, but also identify IL-10 as a potent stimulus of porcine NK cell cytotoxicity.

## Discussion

NK cells are crucial players in the elimination of both virus-infected and malignant cells. Increasing evidence indicates that stimulation of NK cell activity may be a promising strategy in targeting viral diseases and cancer [reviewed in (38) and (39)]. Since swine husbandry is economically important worldwide as well, findings in porcine NK research are not only relevant with regard to their translational potential toward human medicine and research, but may also be implemented in swine industry.

β-glucans have potent immunomodulatory properties [reviewed in (40)]. For example, they are able to alter the gut microbiota and immune system of pre-weaned piglets when supplemented to the feed (23). Furthermore, in-feed supplementation of β-glucans after weaning led to a reduced susceptibility to F4^+^ ETEC infection *in vivo* (41). Hence, β-glucans are looked at as an alternative to in-feed antibiotics to preserve animal health in commercially farmed pigs and as novel health promotor and therapeutic tool in human (para)medicine. Although the effects of β-glucans on immune cells have been investigated in pigs (11) and humans (42), their effects on NK cells remain largely unclear. One report suggested a direct effect of barley β-1,3/1,4-glucans on human NK cell cytotoxicity toward K562 cells (20). However, in that study, NK cells were not FACS-purified and a potential contribution of contaminating cell populations can therefore not be excluded. On the other hand, another study claimed that both zymosan and a particulate yeast β-1,3-glucan showed a direct and dose-dependent inhibition of human NK cell cytotoxicity. In this study, NK cells were also not FACS-purified and β-glucan concentrations were represented as relative particle:NK ratios, which complicates comparisons (22). Here, we aimed to elucidate whether β-glucans directly stimulate FACS-purified NK cells, using porcine primary NK cells and two different types of β-1,3-glucans, Macrogard and Curdlan.

The two tested β-glucans were unable to modulate NK cell cytotoxicity in a direct manner. This observation was also supported by the finding that the major β-glucan receptors, Dectin-1 and CD11R3, appear to be largely absent on porcine NK cells. Therefore, we conclude that there is no evidence for direct effects of β-glucans on NK cell cytotoxicity, at least in pig.

β-glucans have nonetheless been shown to activate innate immune cells such as monocytes, macrophages and neutrophils, including increased cytokine production by these cells (11–13). However, it was unclear if and how such increased cytokine production could alter NK cell cytotoxicity. We found that β-glucan treatment of PBMC resulted in a monocyte-dependent production of secreted factors that enhanced NK cell cytotoxicity *in vitro*. This is in line with a report describing indirect increased cytotoxicity of human NK cells after a 4-day stimulation of PBMC with Lentinan, a β-1,3/1,6-branched glucan (21).

By depleting potentially involved cytokines from the supernatant, we could confirm the pivotal role of IL-10 in the activation of porcine NK cells upon β-glucan priming of monocytes. Although traditionally classified as an immunosuppressive cytokine, IL-10 has been described as an enhancer of NK cell cytotoxicity in human and mouse (43–47). Furthermore, IL-10-mediated increased NK cell cytotoxicity has been suggested to facilitate antigen uptake from dead cells by antigen presenting cells and thereby enhancing the cross-talk between the innate and the adaptive immune system during infection (48). Interestingly, Holder and Grant (49) described that cmvIL-10, a virokine homologous to human IL-10 encoded by human cytomegalovirus (HCMV), increased NK cell cytotoxicity *in vitro* as well. The authors could not determine whether this activity could benefit the virus in some way, but it appears to be in stark contrast with the generally acknowledged evasion strategies of herpesviruses, and cytomegaloviruses in particular, to slow down and suppress NK cell activity (50). Moreover, in acute murine cytomegalovirus (MCMV) infection in mouse, inhibition of the IL-10 receptor increased cell death in NK cells leading to a reduced number of cytotoxic NK cells in the spleen and lung. Together with our current data, this reinforces the hypothesis that IL-10 may contribute to antiviral innate immunity mediated by NK cells (51). Our current data that IL-10 increases NK cell cytotoxicity in pig are thus in line with similar observations in human (43–45), and further support the idea that experimental work on porcine NK cells may generate insights that are also relevant toward a better understanding of human NK cell biology (24, 52, 53).

In cancer biology, the exact role of IL-10 is insufficiently clear and both pro-tumorigenic and anti-tumorigenic functions have been described (54, 55). However, the role of indirect NK cell activation in anti-tumor responses and the underlying mechanisms involved are increasingly studied. It has been shown that N-glycans expressed by tumor cells are recognized by tumor-associated macrophages (TAM) through Dectin-1. This recognition leads to induction of Dectin-1 signaling and is critical to the NK cell-mediated anti-tumor innate immunity (56, 57). An *in vivo* study showed that there was a marked enhancement of metastasis of B16 tumor cells in the lungs of Dectin-1-deficient mice as compared to wild-type mice and that this was indeed due to lower NK cell activity. The exact molecules involved in the cross-talk between TAM and NK cells remain to be further characterized, although requirement of direct cell-cell contact was suggested (57). Based on our current results, however, IL-10 might be an interesting candidate molecule to study in this regard.

Taken together, these results show that β-glucans can significantly enhance NK cell effector functions by triggering NK cell-stimulating IL-10 secretion by monocytes. These findings highlight again the broad influence that β-glucans can exert on the immune response and therefore also the potential of these molecules in the development of novel therapeutic strategies, both in human as in veterinary medicine (42, 58). Since administration of β-glucans is generally not accompanied by side effects, further insights into immune modulation and NK cell activation by these molecules may pave the way toward new strategies in vaccine development, anti-viral therapies and possibly cancer therapies.

## Data Availability Statement

The raw data supporting the conclusions of this article will be made available by the authors, without undue reservation.

## Ethics Statement

The animal study was reviewed and approved by Ethical Committee of the Faculty of Veterinary Medicine (EC2017/121).

## Author Contributions

LH, SP, and SD performed the experiments. LH, EC, HF, and BD designed the research, made the figures, wrote the manuscript and analyzed the results. All authors contributed to the article and approved the submitted version.

## Conflict of Interest

The authors declare that the research was conducted in the absence of any commercial or financial relationships that could be construed as a potential conflict of interest.
