# Supplementary material for: β-Glucan-Induced IL-10 Secretion by Monocytes Triggers Porcine NK Cell Cytotoxicity
Source: Front Immunol. 2021 Feb 19;12:634402. doi: 10.3389/fimmu.2021.634402 (PMC7933222; doi:10.3389/fimmu.2021.634402)
Supplement: Supplementary file 1 [file Data_Sheet_1.docx]

Supplementary Material





**Supplementary Figure 1: Increased β‑glucan concentration does not lead to increased NK cell cytotoxicity.** Purified IL-2-primed porcine NK cells were stimulated with Macrogard or Curdlan at different concentrations (ranging from 10 µg/ml to 200 µg/ml) for 2 h and co-cultured with K562 target cells to asses cytotoxic activity. (MG: Macrogard; CL: Curdlan)





**Supplementary Figure 2: Target cell lysis upon β‑glucan stimulation of PBMC is NK-cell mediated.** PBMC and PBMC depleted from NK cells were cultured for 16 h with or without β‑glucan (Macrogard or Curdlan at 10 µg/ml). Both primed cell populations were co-cultured with K562 target cells to assess cytotoxic activity.





**Supplementary Figure 3: β-glucan primed PBMC do not induce IFN-γ production by pNK cells.** PBMC were cultured for 16 h in pNK medium with β-glucan (Macrogard or Curdlan at 10 µg/ml), with a cytokine-mix (IL 2 at 20 ng/ml; IL-12 at 25ng/ml and IL-18 at 100ng/ml) or in control pNK medium. Afterwards, intracellular IFN-γ was detected via flowcytometry.

**Supplementary Figure 4: LPS (10 ng/ml) does not induce IL-10 secretion by PBMC, but does synergistically enhance IL-10 secretion by PBMC upon priming with Macrogard.** PBMC were cultured for 16 h in pNK medium supplemented with LPS (10 ng/ml), MG (10 µg/ml), MG and LPS together or CL (10 µg/ml). Afterwards, supernatant was collected and the IL-10 concentration was determined by ELISA.
